# Supplementary material for: The GLM-spectrum: A multilevel framework for spectrum analysis with covariate and confound modelling
Source: Imaging Neurosci (Camb). 2024 Feb 2;2:imag-2-00082. doi: 10.1162/imag_a_00082 (PMC12224406; doi:10.1162/imag_a_00082)
Supplement: Supplementary Material [file imag_a_00082-supp.pdf]

The GLM-Spectrum:
A multilevel framework for spectrum analysis with covariate and confound modelling.
Supplemental Material

Andrew J Quinn[1, 2†], Lauren Z Atkinson [1], Chetan Gohil [1], Oliver Kohl [1], Jemma Pitt [1], Catharina Zich [3, 4], Anna C Nobre [1, 5] & Mark W Woolrich[1]

1 Oxford Centre for Human Brain Activity, Wellcome Centre for Integrative Neuroimaging, University Department of Psychiatry, Warneford Hospital, Oxford, UK.
2 Centre for Human Brain Health, School of Psychology, University of Birmingham, UK.
3 Department for Clinical and Movement Neurosciences, UCL Queen Square Institute of Neurology, 33 Queen Square, London, UK
4 FMRIB, Wellcome Centre for Integrative Neuroimaging, Nuffield Department of Clinical Neurosciences, University of Oxford, UK.
5 Department of Experimental Psychology, University of Oxford, UK

† corresponding author: a.quinn@bham.ac.uk

A Time-Averaged Periodogram Estimation

We start by reviewing the definition of the established method for windowed periodogram estimation. The discrete Fourier transform (DFT) can be used to map a series of data points from the time domain into the frequency domain. The frequency domain representation is known as a spectrum and describes how the variance in the data is distributed across frequencies according to a linear basis set. The DFT computes the frequency spectrum $Y(f)$ from an input time series of real values at discrete time points $y(t)$.

$$Y(f) = \sum_{t=0}^{N-1} y(t) e^{\frac{-i2\pi ft}{fs}} \tag{1}$$

Where t is a discrete time point, f is a discrete frequency, fs is the sampling frequency in Hz and N is the number of data points. The output, $Y(f)$, is a complex-valued array containing the estimate of the spectrum. In practice, the computationally efficient Fast Fourier Transform (FFT; Cooley and Tukey (1965)) implementation of the DFT is applied by most software packages. We will use the FFT for this section, as it refers to the algorithm that is most commonly used in practice.

The mathematics underlying the FFT works with an infinite time series. However, the data from real measurements are finite. Therefore, the FFT must implicitly assume that the time-limited input data, $y(t)$, repeats infinitely many times. The combination of this repetition and the discrete sampling of the time-series $y(t)$ leads to the frequency output of the FFT being a linearly spaced axis of N frequency values spanning between $-\frac{fs}{2}$ to $+\frac{fs}{2}$. In addition, the discontinuities between repetitions lead to an effect known as spectral leakage, which spreads power contained in one frequency bin to its neighbours. Finally, the computational efficiency of the FFT relies on the input data length N being an integer power of 2. If it is not, then the routine will zero-pad the length of the input up to a power of 2. This padding causes further sharp changes and discontinuities that can lead to spectral leakage.

The impact of the spectral leakage is reduced by applying a tapered window function designed to flatten the data at the start and end of each segment to minimise discontinuities between repetitions. Here, we modify equation 1 to multiply a window function $w(t)$ with the data (point-by-point) during the FFT:

$$Y(f) = \sum_{t=0}^{N-1} w(t) y(t) e^{\frac{-i2\pi ft}{fs}} \tag{2}$$

There is a huge range of possible window functions that provide different profiles of spectral leakage and sensitivity. The Hamming and Hann windows (used by MatLab's 'pwelch' function[1] and SciPy's 'scipy.signal.welch' function [2], respectively) are two commonly applied options which offer reasonable narrowband resolution. More advanced tapering can be applied using discrete prolate spheroid sequences (DPSS) to create a Multi-Tapered Spectrum estimate (Prerau et al., 2017; Thomson, 1982).

Y(f) is complex-valued, with its real and imaginary parts reflecting the sine and cosine components of the Fourier transform. The phase and magnitude of each frequency component can be computed from these complex values, though spectrum analyses most commonly use the magnitude. We can take the absolute value of $Y(f)$ to

[1] https://www.mathworks.com/help/signal/ref/pwelch.html
[2] https://docs.scipy.org/doc/scipy/reference/generated/scipy.signal.welch.html

create a *magnitude spectrum* $S_y(f)$.

$$S_y(f) = |Y(f)| \tag{3}$$

Similarly, a power spectral density (sometimes just called a power spectrum) can be calculated by taking the squared absolute value normalised by the data length:

$$P_y(f) = \frac{|Y(f)|^2}{N} \tag{4}$$

$P_y(f)$ is a real-valued estimate of the spectral density of a signal, sometimes known as a periodogram. This periodogram provides a relatively simple spectrum estimation but has a further shortcoming. Only one estimate for the power at each frequency is calculated, irrespective of the length of $y(t)$. This means that we have no information about the variance around that estimate, and the estimate does not improve if we include more data.

A better estimator is the Time-Averaged Periodogram, introduced by Maurice Bartlett (Bartlett, 1948, 1950) and refined by Peter Welch (Welch, 1967). These methods are commonly used and ensure that the noise level in the periodogram reduces as the length of the input data increases by a method of ensemble averaging (at the expense of information about low frequencies). This splits the input into a set of $k = 1, 2, \ldots K$ segments (i.e., time windows) each containing $t = 1, 2, \ldots T$ samples and computes the FFT of each to produce a short time Fourier transform (STFT):

$$S_y(f, k) = \sum_{t=1}^{T} w(t) y(t, k) e^{\frac{-i2\pi f t}{fs}} \tag{5}$$

The input for each FFT is now the k-th segment of the continuous input $y(t)$, which we denote with $y(t, k)$. The output matrix $Y(f, k)$ contains the STFT, which describes how the spectrum changes in power across the K segments. A time-varying magnitude spectrum can be computed by taking the absolute value of the STFT.

$$S_y(f, k) = |Y(f, k)| \tag{6}$$

Similarly, a time-varying power spectral density is computed from the squared absolute of the STFT.

$$P_y(f, k) = \frac{|Y(f, k)|^2}{N} \tag{7}$$

Finally, the time-averaged periodogram is then the average of the time-varying power spectral density across segments. If the previous computations included the windowing function $w(t)$ and overlapping time segments, then this is Welch's power spectral density estimate (Welch, 1967).

$$Pwelch_y(f) = \frac{1}{K} \sum_{k=1}^{K} P_y(f, k) \tag{8}$$

Welch's time-averaged periodogram now has the property that the noise level of the estimate decreases with increased data length, since more input data provides a larger number of segments for the central averaging step. It is still an imperfect estimator that has been subject to criticism (Prerau et al., 2017; Thomson, 1982) but it is practical, straightforward to compute, and in wide use across science and engineering.

The real-valued power spectrum may additionally be scaled by a log-transform to produce a log-power spectrum $log(P_y(f,k))$. This can be desirable as the power spectrum is strictly positive and tends to have a strongly non-Gaussian distribution, whereas the log-power spectrum is not strictly positive and tends to have a more Gaussian distribution (See supplemental section C).

Several key parameters must be set by the user when computing a spectrum in this way. These values affect the range and resolution of the spectrum and must be chosen with care. Briefly, there are three main considerations. Firstly, longer segment lengths (T) will increase the frequency resolution (number of bins per Hertz). Secondly, faster data sampling rates allows higher frequencies to be estimated (by increasing the Nyquist range). Finally, increasing the length of the input time-series for a given segment length will increase the number of segments in the average, reducing the impact of noise. These choices are discussed in full in Supplemental section B.

B Parameter settings and resolution in periodograms

When estimating power spectra with a time-averaged approach such as Welch's Method, three parameters are of particular interest: the sampling rate, the length of the window in which the data will be divided, and the length of the data. Changing these parameters can affect the resolution of the spectrum and how many segments are included in the average.

Segment Length. The length of the time segments that the data is divided into influences the resolution of the underlying FFT result. In general, more frequencies are estimated from longer time segments. So, increasing window length whilst holding other parameters constant will result in a larger number of frequency bins in the final spectrum estimate. The resolution in Hz can be computed by dividing the segment length by the sample rate of the data

$$\delta f = \frac{N}{fs} \tag{9}$$

For example, a time series with 512 data points sampled at 128Hz would have a frequency resolution of 4Hz. Similarly, the lowest frequencies that can be reliably extracted from the time-series are also dependent of the window length. Frequencies that are slower than the window period cannot be extracted. In general, it is recommended to estimate power of an oscillatory signal across several cycles to reduce the signal-to-noise ratio of the estimation (Cohen, 2014). Therefore, increasing the window size will assess lower frequencies more reliably (Figure 1A).

There is an additional subtlety in some implementations of the FFT that means that the data length does not necessarily equal the length of the computed FFT. For example, the scipy implementation of Welch's method contains parameters for segment length (nperseg) and FFT length (nfft). These are assigned equivalent values by default, but if they are different to the nfft parameter should be used instead of N to compute the frequency resolution.

Sampling Rate. The FFT returns estimates at equally spaced frequencies from 0Hz to one half of the sampling rate of the data Nyquist Frequency. The 0Hz component is known as a 'direct current' or DC offset and contains the average of all samples in the segment being analysed. The Nyquist frequency is the fastest observable frequency in the dataset and is one half of the sample rate. As such, increases in the sampling rate, whilst holding other parameters constant, will in result in larger frequency spacing and a lower resolution (Figure 1A). The reason for this is that the same number of frequency bins must cover a larger frequency range. In reverse, this means that decreasing the sampling rate while holding the window length constant results in a smaller frequency spacing and higher spectral resolution.

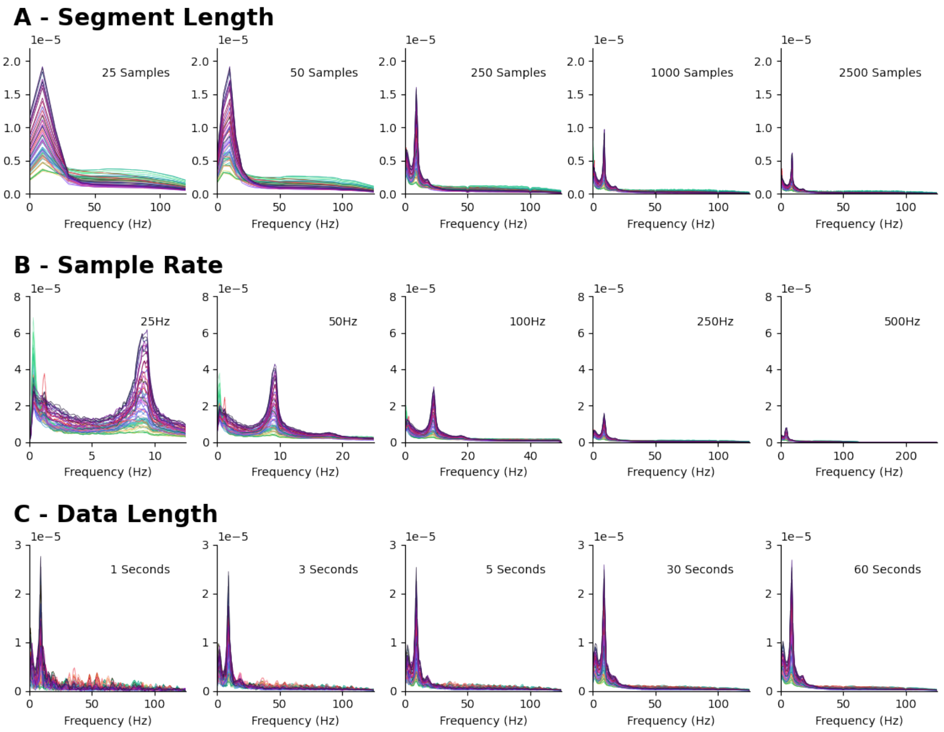

Figure 1. Influence of parameter choice on time-averaged magnitude spectrum estimates.

A: The effect of increasing segment length on the spectrum estimate. Longer segment lengths have a higher frequency resolution. **B:** The effect of increasing sample rate on the spectrum estimate. Faster sample rates have higher Nyquist frequencies and lower frequency resolution. **C:** The effect on increasing the length of the analysed data set. Longer datasets have more segments contributing to the central average and are less affected by noise.

Importantly, increasing the frequency spacing is not always better because smaller bins might result in a noisier estimation of the power spectrum (Figure 1B). Small variations in the (intrinsic) frequency of the oscillatory signals - that are in most cases not of particular interest - might be represented by power in various of these small frequency bins whereas larger frequency bins smooth across these smaller, less relevant variations. This results in a smoother power spectrum.

Length of the overall time-series. The length of the data determines the number of windows in which the time course is divided by Welch's Method before averaging across power spectra. A higher number of windows results in averaging across a larger number of power spectra which in turn results in a better signal-to-noise ratio of the overall power estimation (Figure 1C). In general, considering more data is preferable because the average of a larger number of window-power spectra should result in an estimation closer to the true average power spectrum according to the law of large numbers. Importantly, dynamic fluctuations in oscillatory power over the time course might introduce meaningful variations in the mean power spectrum that cannot be accounted for by Welch's Method and result in more complex, and less peaky power spectra.

C STFT Data Distributions

The general linear model used here expects the dependent variable (the time-varying spectrum) and residuals to follow a Gaussian distribution. Whilst small deviations are permissible, the model fit and any subsequent statistics may be invalid if either is strongly non-Gaussian. This is a concern for the GLM-Spectrum as power values in a standard PSD tend to be non-Gaussian; they are strictly positive and squared (see equation 7). Figure 2A shows an example power spectrum for a single example subject. The second column shows a strongly skewed distribution of power values over time segments for a single channel and frequency. This skew persists into the distribution of residuals as well, indicating that the model assumptions are likely to be violated for when using PSD estimates as the dependent variable in a GLM.

In this paper, we fix this violation by using the magnitude spectrum rather than the power spectrum. Whilst the magnitude spectrum is less commonly used and has less clear mathematical properties [3], though the distribution of magnitude estimates is more Gaussian than for power estimates. Figure 2B shows an example magnitude spectrum, its data distribution and its residual distribution. All are better distributed than the PSD example in Figure 2A. Another option would have been to use the log-power spectrum, which also has a well-behaved Gaussian distribution Figure 2C.

Here, we restored the GLM model assumptions through a data-transform however a more general solution could be to use a model that is robust to different data and residual distributions. Future work could explore using a Generalised Linear Model which can describe skewed power distributions with an appropriate link function (Nelder and Wedderburn, 1972).

[3] The relation between the sum-square of the time-domain data and the integral of the spectrum does not hold for a magnitude spectrum.

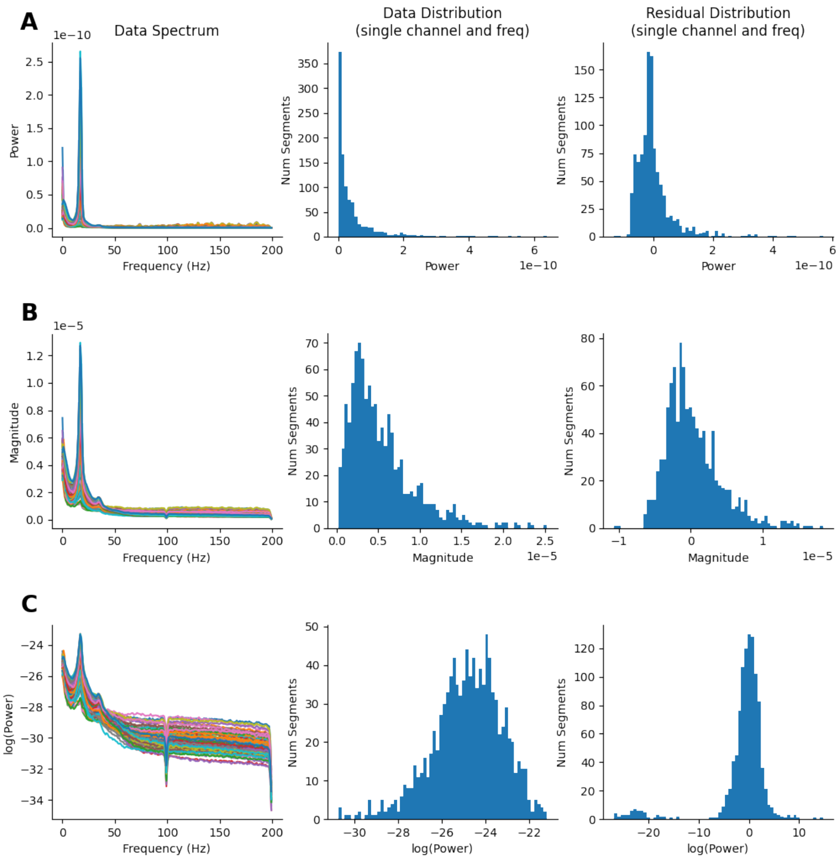

Figure 2. Distribution of the magnitude, power and log-power spectra for an example dataset.
A: The power spectrum across frequency and channels, the distribution of power values over time segments and the distribution of residuals after fitting a GLM-Spectrum **B:** As A, for the magnitude spectrum. The data and residual distributions are substantially more Gaussian in shape. **C:** As A for the log-power spectrum. The data and residual distributions are more Gaussian than the power and magnitude spectra.

D Fast standard-error estimation.

The slowest computation in the GLM-Spectrum is obtaining the standard error of the parameter estimates, known as varcopes. This involves large matrix multiplications that are typically repeated across many tests. However, only the diagonal output is used in the eventual varcope estimate. Standard computing approaches will evaluate every single cell within these matrices even though majority contribute to the off-diagonals and are eventually discarded. This is not a large computational expense for single tests but quickly becomes prohibitive when computing large numbers of test together in matrix form. We use the numpy implementation of Einstein summation conventions (Numpy.Einsum — NumPy v1.23 Manual, n.d.)(https://numpy.org/doc/stable/reference/generated/numpy.einsum.html) to compute the most efficient computational path for the varcope evaluation. In practice, this avoids carrying out intermediate multiplications which would eventually be dropped when extracting the diagonal at the final step. The code for the standard 'DotDiag' approach and the new Einsum approache is as follows:

We explore the difference this makes to computation time by running 100 simulated GLMs with each method for each of four datasets. The first dataset was a single GLM that might represent a single channel and frequency bin. The second is a full GLM-Spectrum across 100 frequency bins whilst the third and fourth were GLM-Spectra across 60 or 204 channels, representing common EEG and MEG data sizes. The GLM was computed 100 times for each case and the results are summarised in Figure 3. The computation was equally fast for both methods in the single-test case but the einsum approach can lead to a 1000x speed up in computation time in the larger datasets. This is convenient for in any case but is essential for making non-parametric permutation statistics of GLM-spectra practically feasible.

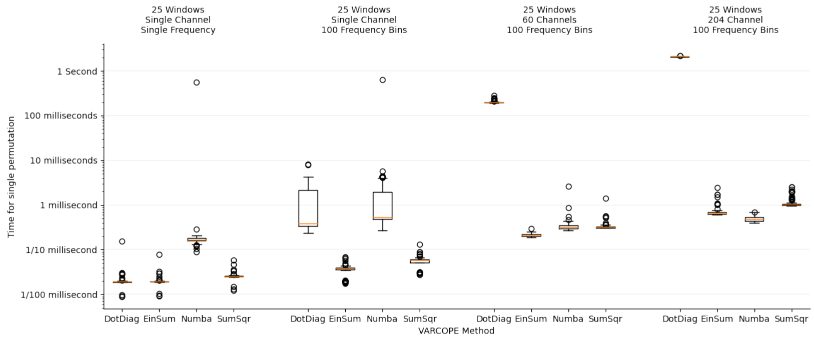

Figure 3. Computation times for varcope estimation across different data sizes with a standard or einsum based method. Each data size and method was computed for random data 100 times and the distribution of its timing shows as a boxplot.

E GLM Design Efficiency

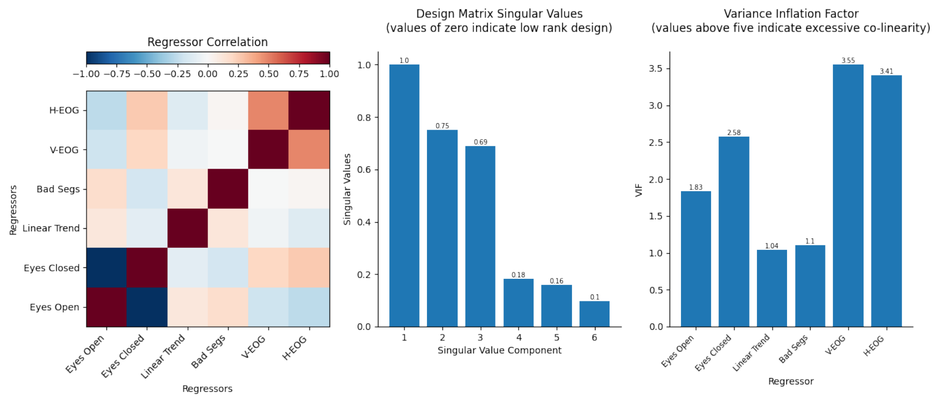

Figure 4. The regressor correlations, singular-value spectrum and variance inflation factors for the first-level GLM-Spectrum design matrix for a single dataset. This analysis indicates that there is some indication of co-linearity but not at a problematic level as the smallest singular-values do not reach zero, and the variance inflation factors do not exceed 5.

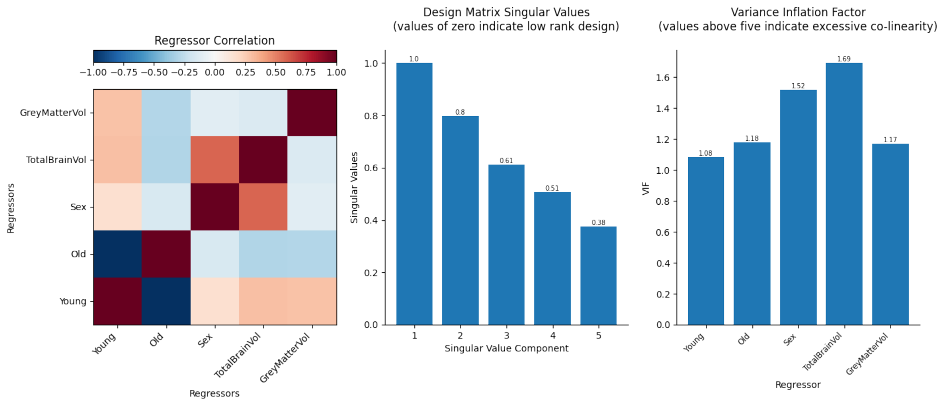

Figure 5. The regressor correlations, singular-value spectrum and variance inflation factors for the group-level GLM-Spectrum design matrix. This analysis indicates that there is some indication of co-linearity but not at a problematic level as the smallest singular-values do not reach zero, and the variance inflation factors do not exceed 5.

F Confound Regression

Figure 6A shows an example design matrix for this case. The first regressor is a constant vector of ones and a second regressor tracks the time segments in which the artefact occurs. In isolation, the constant regressor models the data mean (including the time periods that the artefact occurs), but this changes when it is alongside a regressor with non-zero mean in the same model. The constant regressor now models the intercept; this is the expected value of the data when the artefact regressor is zero (Figure 6B – contrast 1 'Intercept'). In turn, the artefact regressor can be interpreted as the difference between the value of the intercept and the mean of the segments indicated in the artefact regressor (Figure 6B – contrast 2 'Artefact Effect'). We can recover the mean of the artefact segments by summing the parameter estimates for both regressors (Figure 6B – contrast 3 'Artefact Mean').

To illustrate the quantities estimated by the simple model and the confound regression, we generate a simulation of 128 data points centred around a 'true' mean of 1 with a small number of outliers centred around 4 (Figure 6C). The simple mean estimated by the 'mean-only' design is biased towards the outlier observations. In contrast, the confound regression quantifies the 'true mean' of 1 in the intercept term as the artefact regressor describes the effect of the artefact. In a real data example, this design could describe the mean spectrum of a resting-state EEG recording whilst accounting for a set of 'bad segments' annotations identified during pre-processing. This both provides an estimate of any 'artefact effect' and linearly removes its influence from the estimate of the mean term.

We can also specify artefact regressor with the mean removed, i.e. with zero means. Counter-intuitively, the interpretation of the regression parameter estimate is unchanged; whereas the interpretation of the constant regressor changes from modelling the intercept (as in Figure 6B) to modelling the mean over all time points.

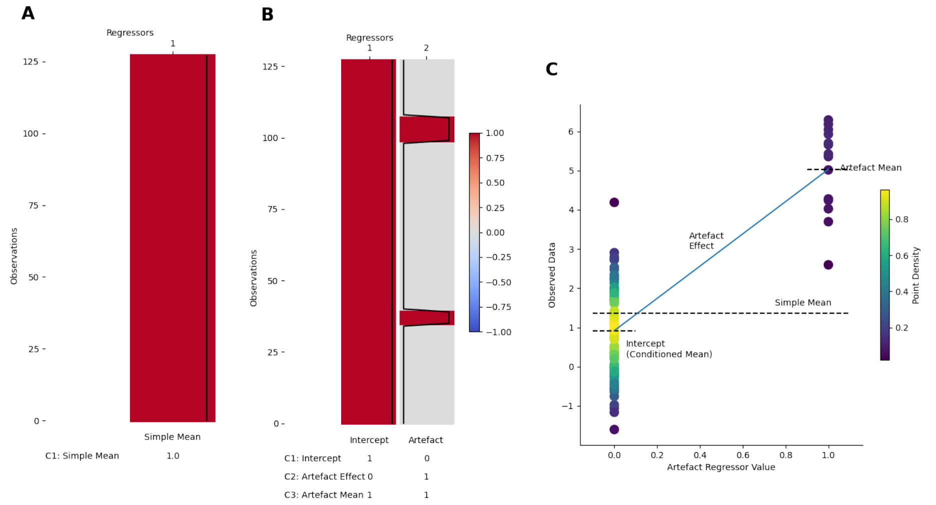

Figure 6. GLM-Spectrum design matrix for confound regression. A: A GLM design with a single regressor and contrast modelling a simple mean of the observed data **B:** A GLM design with two regressors and three contrasts illustrating confound regression. The first regressor is a constant term and the second is a sparse, non-zero mean artefact regressor indicating data observations that are possible artefacts. Three contrasts isolate each regressor individually (intercept and artefact effect) and their sum (artefact mean). **C:** Simulated data observations and the quantities estimated by the GLM designs in A and B. The first design estimates the simple mean overall data, though this is heavily influenced by the possible outlier points. The second design models an intercept and an artefact effect. The intercept can be thought of as the mean where the artefact regressor equals zero, and the artefact effect is the distance between the intercept and the artefact mean. Finally, the absolute artefact mean can be reconstructed by the sum of the two parameter estimates as shown in B contrast 3.

G Effect of Confound Regression on the Open<Closed Contrast

Adding covariate and confound regressors to a model can affect the other parameter estimates and contrasts. This effect is particularly strong when there are correlations between any of the new and existing regressors. For example, the current resting-state recordings interleaves periods of eyes-open and eyes close rest so it is likely that eye movements will increase in the eyes-open periods. This correlation means that a some eye movement related effects might leak into contrast between the eyes-open and eyes-closed conditions unless they are explicitly modelled.

Here, we illustrate this with the group-level average t-spectra for the first-level eyes-open> eyes-closed contrast. This is shown for two different first-level models; a reduced model with no eye movement covariates and a full model with first-level confound modelling. There is a large difference in low frequencies in frontal sensors though the t-spectra are similar in posterior and occipital sensors. Without first-level confound modelling, a large frontal effect can be seen between 1Hz and 4Hz with t-values peaking around 5 (Figure 7A). This is strongly attenuated with the additional confound modelling at the first-level (Figure 7B) which reduces the open > closed contrast t-values in frontal, low frequency regions by around 4.

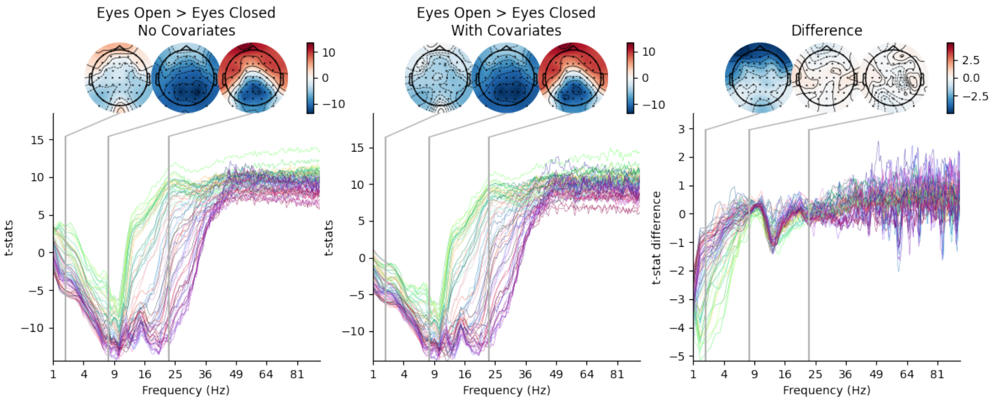

Figure 7. Effect of first-level confound regressors on group-level eyes-open > eyes-closed contrast. A: A t-spectrum of the contrast between eyes-open and eyes-closed resting-states with no covariate or confound regressors. **B:** A t-spectrum of the same contrast as A from a GLM with an additional confound regressor modelling V-EOG activity. **C:** The difference between A and B.

References

Bartlett, M. S. (1948). Smoothing periodograms from time-series with continuous spectra. *Nature*, 161(4096):686–687.

Bartlett, M. S. (1950). Perioidogram analysis and continuous spectra. *Biometrika*, 37(1-2):1–16.

Cohen, M. X. (2014). *Analyzing Neural Time Series Data*. The MIT Press.

Cooley, J. W. and Tukey, J. W. (1965). An algorithm for the machine calculation of complex fourier series. *Mathematics of Computation*, 19(90):297–301.

Nelder, J. A. and Wedderburn, R. W. M. (1972). Generalized linear models. *Journal of the Royal Statistical Society. Series A (General)*, 135(3):370.

Prerau, M. J., Brown, R. E., Bianchi, M. T., Ellenbogen, J. M., and Purdon, P. L. (2017). Sleep neurophysiological dynamics through the lens of multitaper spectral analysis. *Physiology*, 32(1):60–92.

Thomson, D. (1982). Spectrum estimation and harmonic analysis. *Proceedings of the IEEE*, 70(9):1055–1096.

Welch, P. (1967). The use of fast fourier transform for the estimation of power spectra: A method based on time averaging over short, modified periodograms. *IEEE Transactions on Audio and Electroacoustics*, 15(2):70–73.
